# ‘I’m Gonna Tell You about How Mrs Rona Has Affected Me’. Exploring Young People’s Experiences of the COVID-19 Pandemic in North East England: A Qualitative Diary-Based Study

**DOI:** 10.3390/ijerph18073837

**Published:** 2021-04-06

**Authors:** Stephanie Scott, Victoria J. McGowan, Shelina Visram

**Affiliations:** 1 Population Health Sciences Institute, Faculty of Medical Sciences, Newcastle University, Sir James Spence Building, Royal Victoria Infirmary, Newcastle upon Tyne NE1 4LP, UK; victoria.mcgowan@newcastle.ac.uk (V.J.M.); shelina.visram@newcastle.ac.uk (S.V.); 2 NIHR Applied Research Collaboration North East and North Cumbria, St Nicholas’ Hospital, Jubilee Road, Gosforth, Newcastle Upon Tyne NE3 3XT, UK

**Keywords:** young people, mental health, education, COVID-19, qualitative, diaries

## Abstract

Children and young people risk being ‘disproportionately harmed’ by the COVID-19 pandemic. Whilst an evolving body of literature focuses on the impact of the pandemic on the mental health and wellbeing of children and young people, less attention has been paid to the collection of qualitative, exploratory data. The aim of this study was to examine young people in North East England’s experiences of COVID-19 and associated control measures. Flexible, qualitative diaries were collected with 31 young people aged 13–17 for six weeks between July and October 2020. Diary extracts were curated using Instagram Direct Messaging (DM), email and text messaging. At the end of this study, participants took part in a follow-up interview (conducted by telephone or Zoom), asking them to reflect on their diary entries. Thematic analysis of diaries and interviews yielded three central themes: (1) impacts upon mental health and emotional wellbeing; (2) disruptions and changes to education and school life; and (3) frustration, burden and responsibility. These findings highlight acute mental health impacts (loneliness, isolation, anxiety) as well as longer-term repercussions from disrupted education (missed parts of curriculum, home schooling, cancelled exams, periods of isolation) on young people aged 13–17 as a result of the COVID-19 pandemic.

## 1. Introduction

Since March 2020, countries worldwide have implemented strict national and localised controls on movement in order to reduce the spread of COVID-19 and ease pressure on key services. UK measures have included the closure of schools, colleges and universities; bans on social gatherings; the closure of retail outlets; and strict limits on contact with specific settings or population groups [1]. Such measures have reinforced existing health inequalities and, in some cases, increased them [2]. This phenomenon has been described as a ‘syndemic pandemic’, where COVID-19 acts synergistically with—and exacerbates—existing socio-economic, geographical and ethnic inequalities [3], emphasised in the most recent Marmot review, which urges us to ‘Build Back Fairer’ post-pandemic [4].

Children and young people (CYP) are at particular risk from long-term repercussions of the COVID-19 pandemic [2]. The Global Survey on Youth and COVID-19, conducted with over 12,000 young people aged 18–29 in April and May 2020 revealed the impact of COVID-19 to be systematic, deep and disproportionate, with particular consequences for young women, younger youth and those in lower-income countries [5]. In the UK, this message has been reinforced by Scientific Advisory Group for Emergencies (SAGE), who have warned that those aged 7–24 years risk being ‘disproportionately harmed’ by the pandemic. For many CYP, this includes psychological impacts relating to isolation and disrupted educational provision and attainment at critical timepoints [6,7]. For others, increased risks are faced at home due to parental substance misuse, domestic violence or food insecurity [8,9,10], as well as longer-term repercussions relating to the wider social determinants of employment and lifetime earnings [11].

An evolving body of research focuses on the impact that the pandemic and subsequent lockdown restrictions have had on CYP’s mental health and wellbeing [12]. In Ireland, children and adolescents have experienced feelings of social isolation, depression, anxiety and increases in maladaptive behaviour [13]. In the UK, such impacts are likely to be particularly pronounced due to sustained periods of lockdown. Thus, the 2021 Princes Trust Youth Index demonstrated that one in four 16–25 year olds (26%) feel ‘unable to cope with life’, rising to 40% amongst those not in education, employment or training [14] whilst Vizard et al. (2020) identified that, among young people in England aged 5–16, the prevalence of probable mental disorder has increased from one in nine (10.8%) to one in six (16.0%) [15]. Likewise, Dewa et al. (2021) found that, for those aged 16–24, mental health has significantly worsened since lockdown, and dysfunctional coping strategies, such as substance misuse, sleep problems, and self-blame are associated with this [16].

Several oral history projects and qualitative studies have began (or are about to embark upon) projects using serial or longitudinal qualitative methods to explore CYP’s experiences of the pandemic [17,18]. Based predominantly on in-depth interviews with young people aged 14–18 in the UK, Italy, Singapore, and Lebanon, findings from an initial cycle of action research by Day et al. (2020) suggest that young people felt marginalised through the early stages of the pandemic due to lack of access to health care, education, and other services. Young people also felt overlooked in decision making about how the pandemic is handled. A smaller number of, as yet largely unpublished, research studies have used qualitative diaries to document COVID-19, such as ‘CoronaDiaries’ [19] and ‘Covid Realities’ (https://covidrealities.org/, accessed on 12 February 2021), as well as ‘Coronavirus Diaries’, implemented by Britain Thinks, which asks 50 people across the UK to complete a weekly poll tracking their mood, views on the government and media consumption (https://britainthinks.com/news/britainthinks-coronavirus-diaries, accessed on 12 February 2021). Nevertheless, these studies have predominantly captured the experiences of adults or families. To our knowledge, just one of the studies outlined above has collected diaries directly with young people, as part of a youth-led, ethnographic action research project [17]. Findings from diaries are as yet unpublished. Further, this is an international study with participants from the UK combined with young people residing in Italy, Singapore, and Lebanon.

Thus, our findings extend this developing area of work by focusing specifically upon mid-adolescents (aged 13–17) in one English region (the North East), thus providing temporal relevancy to the policy context and restrictions in place within the UK. Repercussions of the pandemic will be felt geographically. The North of England’s economy has been hit harder than the rest of the country during the COVID-19 pandemic, with inequalities between the ‘Northern Powerhouse’ (North East, North West, Yorkshire & Humber) and the rest of the country exacerbated [20]. These bleak findings were also reinforced in a recent survey focusing on 14–30 year olds in North East England [18].

Therefore, the aim of this study was to use diary-based qualitative methods to explore young people in North East England’s experiences of the COVID-19 pandemic and associated control measures. We set out to examine: (1) the impact of COVID-19 on young people’s physical and mental health and wellbeing; (2) young people’s coping mechanisms during periods of restriction; (3) the impact of ‘fractured’ or ‘interrupted’ experiences of schooling; (4) young people’s worries and hopes about their future; and (5) young people’s expectations of a post-pandemic ‘normal’. To our knowledge, this is the only published study (to date) to use written and/or visual, arts-based diaries over a prolonged period of time to document UK young people’s journeys in and out of lockdown over the course of the pandemic.

## 2. Materials and Methods

### 2.1. Sampling and Participant Information

Data were derived from a broader longitudinal project focused on understanding young people’s experiences of pandemic restrictions and the impact of restrictions on their mental health, wellbeing and education. This paper reports findings from the first exploratory phase of data collection, in the immediate aftermath of the first national lockdown. All participants resided in North East England, which has been marked as an area of ‘high COVID-19 risk’ and subject to strict social distancing restrictions since the implementation of localised tier systems at the end of summer 2020. We focused efforts on the recruitment of young people in areas of deprivation. Participants were recruited from youth and community organisations, detached youth work schemes, and regional charitable and third sector organisations, as well as via social media. We also distributed information about this study through university and funder networks, as well as our personal and professional networks. Young people were purposively sampled according to age (at date of recruitment), gender and socio-economic status (SES). SES was classified using the Index of Multiple Deprivation (IMD) quintiles (where 5 is ‘most deprived’) and determined by participant postcodes. Sample size was ultimately guided by: the breadth and focus of the research aims and objectives; the demands placed on participants; the depth of data likely generated; pragmatic constraints; and the analytic goals and purpose of the overall project [21]. 

All participants took part in a short ‘getting-to-know-you’ telephone conversation with the lead researcher (S.S.) at the beginning of this study. During this conversation, young people completed a digital consent form, a copy of which was sent to them for their records; verbal consent was also sought from a parent or guardian for those under 16. Participants provided demographic information (age, gender, ethnicity and postcode) and completed the Warwick–Edinburgh Mental Wellbeing scale (WEMWBS), validated for use with young people [22,23]. At the end of this study, all participants re-completed the WEMWBS and took part in a follow-up interview (conducted by telephone or Zoom). All interviews took place between September and November 2020. Average interview length was approximately 15 min. All interviews were conducted by the lead researcher (S.S.), who, at the time of this study, was a female mid-career researcher (educated to PhD level), aged 35 years, highly trained and experienced in qualitative interviewing and analysis, particularly with young people. Participants received a £20 gift voucher to compensate them for their time, as well as a £10 voucher to offset data/electricity costs.

### 2.2. Data Collection Techniques

**Figure 1:**
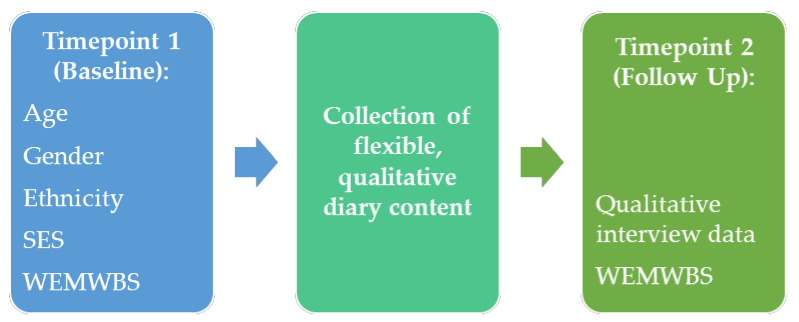
Type of data collected at each study timepoint.

Digital, qualitative diary extracts were solicited specifically for the purpose of research with 31 young people aged 13–17 for six weeks between July and October 2020; followed up by a semi-structured interview (see Figure 1 below which summarises data collected at each study timepoint). This time period allowed us to explore young people’s thoughts and feelings following the end of ‘Lockdown 1’, during summer (where national restrictions were relaxed significantly in the UK) and upon their return to school. During follow-up interviews, we explored: (1) diary extracts received over the course of this study; (2) whether there was anything important to them that they had not shared; (3) how they felt COVID-19 would impact on the next six months and beyond.

An emerging evidence base suggests that diaries can yield rich, complex layers of understanding, particularly with marginalised groups or where topics may be difficult to discuss face to face [24,25,26]. Diaries may also facilitate prolonged engagement with research participants, where people may have difficulty articulating themselves, enhancing rigour, quality and trustworthiness of data collected [27,28]. For young people particularly, diaries offer a way to express themselves, perhaps with less embarrassment, or fewer feelings of being judged, than in interview scenarios [29]. They may also offer young people a greater degree of control in the research process [30]. Ontologically, use of diaries aligned both with our focus on exploring the unfolding nature of the crisis, one which could not be captured in a single narrative [31]; as well as with a feminist ethics of care and justice, which emphasises the need to ensure that experiences can be sensitively captured and explored [32], and which suggests that diaries have the potential to be cathartic or therapeutic for participants [33].

Like Herron et al. [31] and Breheny et al. [34], we employed a flexible approach to the format of diary content, allowing young people to choose how they wished to narrate their experiences. Therefore, a range of media was collated including text, photos, audio, and drawings, using a combination of email, text messaging and Instagram. Project pages with the study name ‘Lockdown Life North East’ were set up on both Instagram and Facebook that participants could choose to ‘follow’, with private diary extracts collected using Direct Messaging (DMs). Participants were not obligated to engage daily; and the lead researcher (SS) encouraged regular contact by sending open and neutral prompt questions, probes and posts on the project Instagram page (for example: ‘is there anything that you’d like to share or tell us about today’). Use of digital platforms such as Zoom, Teams, as well as extended use of telephone interviews and social media platforms, arguably represent ‘the new normal’, particularly during and post-pandemic [35,36]. Young people are increasingly used to communicating in these spaces [37]. A growing literature base indicates that collecting data in this way provides rich, multi-media insights into the world of young people, not readily accessed through other methods [29,38].

### 2.3. Data Coding and Analysis

Given the exploratory nature of this study, and the rarity of a global pandemic on this scale, our analysis was not wedded to a pre-existing health or sociological theoretical framework. We have taken an inductive approach to theory building, guided by the data and a constantly developing and evolving pandemic response. Data collection and analysis took place concurrently, in order to continually re-evaluate emergent findings, and to allow probes and prompts to be tailored each week. All young people consented to their follow-up interview being audio recorded. Interviews were transcribed verbatim, with observational field notes maintained in a research diary. Data from email, text messaging and social media platforms were manually downloaded (copy-pasted) and combined with interview transcripts for analysis. Primary data comprised written material (diary and interview extracts), with visual materials used to elicit detailed and located narratives from young people.

Diary extracts, verbatim interview transcripts and field notes were analysed using applied thematic analysis techniques [39], following the principles of constant comparison [40] to enhance internal validity [41]. Our approach to analysis was multi-dimensional, ensuring analysis of both visual and text-based elements of the data. Thematic analysis was completed in two core stages. First, diary extracts were downloaded and interview audio files were transcribed, checked for accuracy and anonymised by the research team. This process also facilitated familiarisation. The lead researcher (S.S.) inductively identified codes in the data which were constantly compared across the diary extracts and interview transcripts and used to identify sub-themes. Data were subsequently coded at three levels (open, axial, and selective) to explore linkages between the data and systematically indexed into an initial coding scheme. Second, regular data analysis meetings were held with co-investigators (S.V. and V.J.M.), at which source data and the developing coding scheme were challenged and refined using a process defined as pragmatic double coding [42]. Here, developing descriptive themes were compared to identify patterns, similarities and differences in the data, and relationships between them elaborated, in order to agree broader, overarching analytical themes, and a consistent interpretation of the dataset as a whole.

### 2.4. Rigour and Trustworthiness

Our approach to data collection, coding and analysis was guided by COnsolidated criteria for REporting Qualitative (COREQ) research [43]. Our full COREQ checklist is shown in Supplementary S1. Several established approaches were also taken to ensure the validity and rigour of the findings [44,45], including development of a coding system, peer review of themes, prolonged engagement and rapport with participants, triangulation of multiple data sources (diary extracts, visual material and interview transcripts), and provision of thick description that recognises the context of data collection, supported by quotes and detailed field notes. Ethical approval for this study was provided by Newcastle University (REF 1916/2666, 15 June 2020). No relationship between the interviewer and participants was established prior to this study. All participants received a study information leaflet, which included details about the interviewer’s credentials and reasons for conducting this study; anonymity was assured.

## 3. Results

### 3.1. Participant Characteristics

Thirty-two young people were initially recruited; one young person changed their mind and withdrew from this study a few days after recruitment, resulting in the analysis of data from 31 young people. Participant characteristics (gender, age, level of deprivation, ethnicity and WEMWBS category) are presented in Table 1 below. Participants were predominantly white British individuals (94%), reflecting the demography of the study area. Using the crude measure of an IMD quintile of between 1 and 5 (where 5 is most deprived), 39% of participants were of low SES (quintile 4 and 5 combined). At the beginning of this study (defined as timepoint 1), 13% of those recruited into this study were categorised as ‘possible depression’ and 16% were categorised as ‘probable depression’ using WEMWBS. At timepoint 2, possible depression remained the same whilst probable depression increased to 23%.

The restrictions that young people describe in the sections that follow include social distancing, both in a literal (maintaining 2 m distance) and figurative (ceasing or reduction in contact with friends and family members outside of the own household) sense, school closures and remote learning, and localised lockdowns of retail, community and hospitality spaces. Such restrictions interacted, amplified and overlayed in young people’s lives. Analysis of diaries and interviews yielded three central themes: (1) impacts on young people’s mental health and emotional wellbeing; (2) disruptions and changes to education and school life; (3) frustration, burden and responsibility. Each overarching theme consisted of several sub-themes, which are outlined in Table 2, Table 3 and Table 4, reflecting how our coding scheme was indexed into themes. The findings presented below include quotations to provide rich description and faithful accounts of the views and experiences of young people in this study. All data were coded anonymously to ensure that participants were not identifiable from their accounts.

### 3.2. Impacts on Young People’s Mental Health and Emotional Wellbeing

Sub-themes and further supporting extracts for this theme are illustrated in Table 2 below. Life for the young people in our study was complicated or different in ways that they could not have imagined previously, and many did not expect restrictions to be so prolonged. They expressed a sense of loss or grief for pre-pandemic life and things that had previously been taken for granted, such as sports, sleepovers, and time with loved ones:

“*I’m gonna tell you how Mrs Rona has affected me. Before lockdown my life was busy and I liked it that way, always having something to do whether that’s sports, going out or just having fun. Corona came along and you do try and stay positive but I believe in everyone’s case we didn’t think the virus would go on for this long and it would be a short term thing. Months went on, you just felt a bit claustrophobic and like life’s not really real in a way*”—Female, Aged 16, Diary Extract, 24 August 2020.

Young people frequently took a step back to reflect and described this period of their life as ‘surreal’ or ‘the new normal’, with some detailing how they might feel if and when things ‘return to normal’ or what a post-pandemic future will look like:

“*you are gonna have to keep a mask on you, wear your mask, keep your hands sanitised. I think even after this is all over, It’s still going to be even weird, being in a social space, being in town, like with so many people…One thing me and somebody else was talking about, was concerts, who is going to trust going to a concert again, like a proper one*”—Female, Aged 16, Zoom Interview, 2 October 2020.

**Figure 2:**
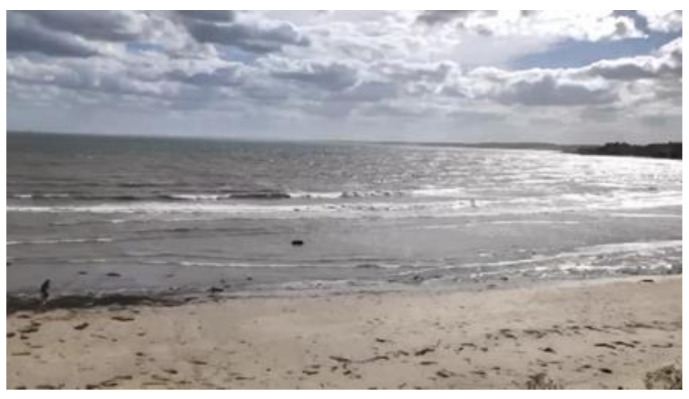
“*This week has been nice calm and relaxing. We went to see my uncle and to the beach hut to get fish and chips*”—Female, Aged 13, Diary Extract, 21 August 2020.

All of the young people in our study experienced emotional repercussions linked to the pandemic. Most young people described feeling lonely, anxious and all of them missed their friends, extended family, social contact and interaction. Several had pre-existing mental health conditions or lived experience of difficulties which were exacerbated during national restrictions. This meant that opportunities for face-to-face contact during Summer 2020, when restrictions were eased, were of huge benefit to young people’s mental health and wellbeing (see Figure 2). As we moved towards Autumn and Winter 2020, most participants again felt isolated and frustrated, both with the unreliability of the weather and with rapid changes in UK restrictions.

“*Today has not been a very good day as I found out that we are being put in a local lockdown. This actually made me really upset as it means I can’t see my family anymore. I found out as soon as I got in from school and my full mood dropped. I went to my grandma and grandads house so I could see them before we went into local lockdown at midnight. It was really sad knowing that this was going to be the last time I saw them for the foreseeable future. I really hope it doesn’t last long and we can go back to how things were really soon*”—Female, Aged 15, Diary Extract, 18 September 2020.

Nevertheless, not all young people experienced lockdown in the same ways. Some described being excited about lockdown, at least initially, with others identifying positive impacts. It provided a change in pace or a break for those where school was a source of anxiety; for others, it strengthened family bonds.

“*I actually enjoyed lockdown because you only had to speak to people that you wanted to. You can’t exactly walk out of a lesson to go walk your dog*”—Male, Aged 14, Telephone interview, 9 October 2020.

**Figure 3:**
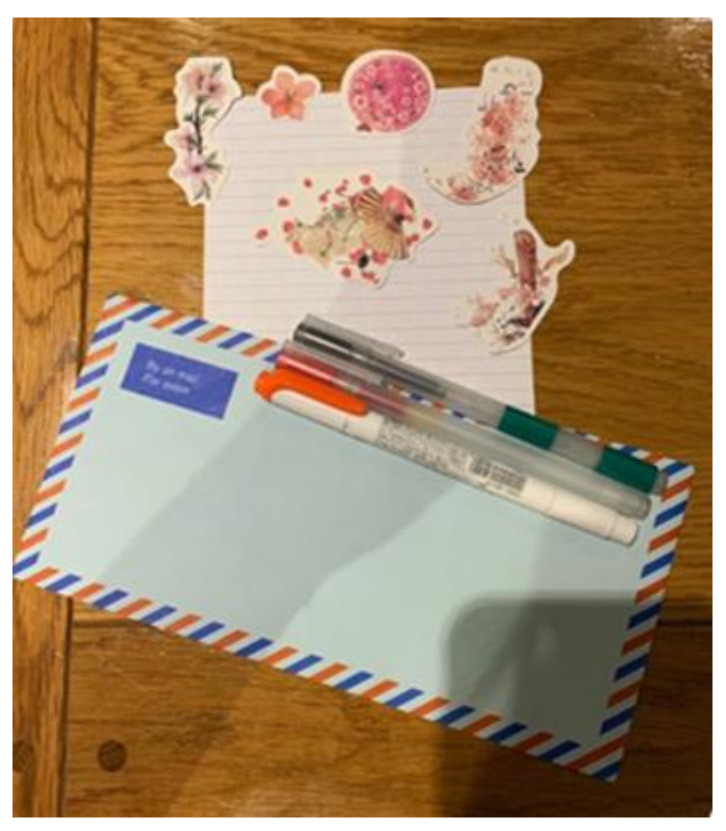
“*So I’ve been writing letters to my friends. A few of them are within walking distance so I just pretend that I’m mailing it by writing their address and drawing a stamp making it look cute. Writing letters has been a fun way to just chat to them in a way we wouldn’t do over the phone or in person because it feels too ‘cringe’…I think it’s just our generation who doesn’t get letters anymore. Whenever you do get a letter, it’s quite exciting…it was kind of like the thrill of it. Of just getting a letter through the post with your name on it.*”—Female, Aged 16, Diary Extract, 1 August 2020 and Zoom Interview 2 October 2020.

Finally, for some young people, keeping busy by maintaining routines and a sense of normality, or even a sense of excitement over relatively small things, in order to break the mundane nature of lockdown, were helpful. For example, young people described trying new hobbies, interests or activities during this time (see Figure 3).

“*When I found out we were going into lockdown I was actually quite excited about the idea—came at a time when school was getting quite stressful (mocks approaching and lots of work). I saw lockdown as an opportunity to sort of reset and take a month or two just to relax. I also get quite down in the winter months, not depressed but less happy i guess, so felt like time with not school would be good. Once we got to about 2 weeks into lockdown I started getting really on edge and found myself getting annoyed and frustrated easily especially with my little brother*”—Male, Aged 17, Diary Extract, 31 August 2020

### 3.3. Disruptions and Changes to Education and School Life

Sub-themes and further supporting extracts for this theme are illustrated in Table 3 below. The collection of diaries began in early summer 2020 and, in initial extracts, many young people reflected back on the first national lockdown in England. Whilst ‘Lockdown 1’ was difficult in many ways for young people in our study, as set out above, for some it was also relatively uncomplicated by school work, particularly those who had exams cancelled. Those that did have school work to complete were able to do so at a pace that suited them. As September grew near, young people began to look towards returning to the school setting, with many articulating mixed feelings about this return. They did want to return to regular face-to-face teaching, see their friends, and resume activities; but reflected acutely on the danger, stresses and strains that resumption signified:

“*I start school again on Tuesday which is annoying and its definitely going to be very different which worries me a bit, particularly because we have to wear face masks not to mention the fact that it is year 11 that I am starting. But I will get to see all of my friends again so that’s good.*”—Female, Aged 15, Diary Extract, 6 September 2020

As life became busier, young people felt the emotional strain of higher workloads and anxiety about school, and the impact that lockdown restrictions could have on their future, were central to young people’s narratives. This was particularly pertinent to those facing educational transitions, for whom GCSE or A-Level exams were subsequently cancelled, and those who recognised that the pandemic may impact those sitting exams in 2021. For those young people, lockdown and the months leading up to exam results day were fraught with uncertainty as, for a long period of time, they were left unsure as to how their results would be calculated.

*“my college experience is being put in jeopardy”*—Female, Aged 16, Diary Extract, 14 September 2020.

Some young people expressed disappointment with being unable to sit exams, and the lack of closure this, and other milestones associated with leaving school such as prom, represents. Some young people felt that they had somehow cheated by getting good GCSE or A-Level grades, even though their eventual grades were a reflection of mock results and the effort they had put in up until lockdown. They felt that, without physically sitting the exam, that their grades were somehow not real or ‘not deserved’. For those starting college, this knocked their confidence and introduced an element of ‘imposter syndrome’:

“*The gov is saying that real exams will go forward no matter what but I reckon coming out of winter the pandemic will be at its worst and I reckon it won’t be good enough to have exams. Personally I want to do the actual exams…I feel like exams give a sort of closure to the school period of my life which i thinks kinda important. I know going from year 12 to 13 felt like there was no real change (as if it were still the same school year as there’s been no defined end so I wouldn’t want to feel like that going into uni. If that makes any sense?*”—Male, Aged 17, Diary Extract, 6 October 2020.

Nevertheless, on a wider level, most participants were pleased and/or relieved to return to school. This aided the loneliness and sense of loss many had experienced, and provided a much-needed return to normality:

“*I am very anxious at the idea of going back into lockdown because when I look back I was extremely lonely and I have really enjoyed being around people again and socialising*”—Female, Aged 15, Diary Extract, 22 September 2020.

Even where excited or relieved, returning to a busy school setting was a shock after six months away, as well as tiring, as young people reported attempting to ‘catch up’ on content that they had missed during lockdown:

“*This week I received about 8 pieces of Homework. It felt quite stressful due to not doing it in a long time, with a due date. The only problem is that I am often quite tired. School has been alright and it’s just getting back to normal which is quite hard. Since I’m starting year 10, it’s my beginning of GCSE. On Thursday I ran out of energy and felt quite low but on Friday I was back to normal*”—Male, Aged 14, Diary Extract, 13 September 2020.

Young people were also daunted by widespread changes at school and new social distancing rules such as one-way systems, bubbles and wearing face masks. Some rules made school life a lot harder for the young people in our study—many spent all day in the same classroom, hot lunches were restricted, and many could not use school computers or interact with friends or their teacher in the same way. They were frustrated and confused about how rules were enforced, particularly when it came to what should be defined as ‘close contact’ and why they can see their friends inside, but not outside, of school:

“*Here’s something I don’t understand about social distancing. In college, I have to sit next to someone in lesson and that’s our “bubble” but when our “bubble” is sat next to each other in our resource centre that’s not allowed at all. It makes absolutely no sense. Our small bus can be full of people with no spacing at all, but I can’t sit next to a friend even when I have a mask on. It’s really confusing and contradicting. Also, how in some areas you can sit next to someone but in other areas its not allowed? I really don’t understand how in some places you can’t mix households with your family, but you can go into a college with 1200 different households and that’s perfectly fine*”—Female, Aged 16, Diary Extract, 7 October 2020.

Finally, school responses to localised outbreaks meant that young people’s learning continued to be fractured or disrupted across September, October and November, with teachers and classmates needing to isolate periodically. This added to the levels of anxiety and stress that many young people in our study were experiencing:

“*First case of coronavirus in my year and over 40 people have to self-isolate for 14 days including some of the teachers. Luckily I haven’t been in contact with the person who has tested positive but there has also been quite a few people sent home with coughs etc. I’m a bit worried but trying not to let it get to me. My maths teacher has to self isolate so I won’t be getting taught properly for 2 weeks which is frustrating*”—Female, Aged 15, Diary Extract, 21 September 2020.

### 3.4. Frustration, Burden and Responsibility

Sub-themes and further supporting extracts for this theme are illustrated in Table 4 below. Young people in our study felt a sense of responsibility or burden for other people’s health as well as their own. They felt that, as a generation and as individuals, they had given things up in order to keep people safe. This led to missed milestones (lockdown birthdays, Zoom proms, not being able to celebrate exam results day with family and friends) and feeling left out. Thus, they expressed frustration, anger, and disappointment, largely due to constant change, uncertainty and lack of control. Young people in our study found themselves having to engage with news updates on an unprecedented scale, and described needing to be constantly prepared and ready to adjust to changing guidelines and restrictions. This was exhausting and meant young people were operating at heightened emotional states.

“*So Boris has changed the rules back to gatherings up to 6 people—I thought that was already the maximum but apparently it was up to 30 at one point. The lack of communication is really confusing, and the contradicting messages is just an absolute joke*”—Female, Aged 16, Diary Extract, 14 September 2020

They tended to direct their frustration and anger in three different directions. First, with the government for setting what they felt were non-sensical rules (such as Eat Out to Help Out, a UK Government policy scheme offering discounts to encourage consumers to eat out and support the hospitality sector which ran during August 2020) or for implementing/easing restrictions at the wrong times (delays to national lockdowns):

“*Quite recently new restrictions have been introduced for Coronavirus: including a curfew at 10:00 and meetings above 6 outside of school banned which has obviously caused a stir in the community. Personally I feel the government are contradicting themselves, going from discounts on meals to get people back in restaurants to reintroducing policies about group size, not to mention the fact that young people aren’t allowed to meet up outside of school despite spending 6 h jammed in crowded corridors together. Although I think some precautions need to be taken, me not being able to see my school friends is pointless and stupid.*”—Male, Aged 14, Diary Extract, 23 September 2020

Second, they levelled disbelief and frustration at rules imposed ‘on the ground’, largely at school, which they felt did not make sense such as not being able to socially distance fully when travelling to and from school (such as on the bus) and not being able to mix with classmates outside of school. Finally, they expressed disappointment and at times anger with those not following rules and restrictions and not taking risks seriously, with some young people describing this as ‘selfish’, suggesting, at times, that this led to young people being unfairly blamed or labelled for spreading the virus.

“*It got to the point where people just wouldn’t ask me out. Which, obviously, fair enough, but it was still quite disheartening. I don’t know if that’s the word, but it was big FOMO, like I definitely felt a lot of FOMO during lockdown, when people would still go out, and then I had made this decision to not go out*”—Female, Aged 16, Zoom Interview, 2 October 2020.

Thus, with the exception of one or two young people who felt restrictions were too heavy handed, young people in our study described high levels of compliance with pandemic restrictions. This reflects findings from other studies focusing on adherence to rules and rule breaking. Whilst frustrated and tired, they suggested that restrictions, such as lockdowns, were necessary in order to keep people safe and worried that attempts to return to ‘normal’ life too quickly would ultimately be harmful:

“*The government is also opening lots of things up then blaming people for socialising—if it’s not safe to socialise, then they shouldn’t be encouraging people to use them. It’s just all annoying.*”—Male, Aged 16, Diary Extract, 15 September 2020.

## 4. Discussion

### 4.1. Overview of Key Findings

Our findings highlight acute mental health impacts (loneliness, isolation, anxiety) as well as longer-term repercussions from disrupted education (missed parts of curriculum, home schooling, cancelled exams, periods of isolation) on young people aged 13–17 as a result of the COVID-19 pandemic. In the sections that follow, we have mapped our findings against the original aims of our study, to illustrate how we met these a priori aims, and how our data advances understanding in each of these areas, reinforced by supporting literature.

#### 4.1.1. The Impact of COVID-19 on Young People’s Health and Wellbeing

Like participants in Demkowicz et al. (2020), young people in our study experienced the removal of ‘normal life’ and lack of social contact on an unprecedented scale, culminating in feelings of uncertainty, loss and grief [46], feelings that have transcended generations during the pandemic [47,48]. The extent and long-term repercussions of this remain largely unknown—whilst there are studies to suggest that confinement of adults is associated with symptoms of depression, increased risk of mood disorders, irritability and stress, evidence among children and young people is anecdotal with limited conclusions [49]. Nevertheless, our qualitative data strongly highlight that the impact of COVID-19 was not the same for everybody, a finding reinforced by WEMWBS data. WEMWBS data were collected from young people at point of recruitment and during follow-up interviews 6-weeks later. Investigating longitudinal quantitative change was not an a priori aim of this study. However, whilst we cannot draw any major inferences from WEMWBS data—the sample is not big enough for this to be statistically significant or to be able to have wider generalisability—it is important to note that, at the beginning of this study (defined as timepoint 1), 61% of those recruited were categorised as having an ‘average’ score of mental health and wellbeing using WEMWBS, reducing to 45% at timepoint 2. However, both those categorised as having ‘high’ mental health and wellbeing and those categorised as ‘probable depression’ increased at timepoint 2. Speculatively, this may demonstrate a ‘splitting of the average’. In other words, for many young people, restrictions as a result of the pandemic were a negative experience; however, for others, institutions such as school and the outside world were possibly the source of their anxiety, factors which were removed during periods of lockdown. Further, the emotional impacts of the pandemic appeared more pronounced in the narratives of female participants, with this reflection again complimented by WEMWBS scores.

This idea is supported by wider literature. Whilst some evidence suggests young people’s mental health has worsened during the pandemic [15,16], others have shown a decreased risk of anxiety and improvements in overall wellbeing [50]. Thus, Widnall et al. (2020) found that, in a survey of 13–14 year olds, compared to pre-pandemic levels, there was an overall decrease in risk of anxiety, and an increase in wellbeing but no large change in risk of depression, with students’ school connectedness increasing overall. Further, the largest improvements in mental health and wellbeing were for students who had poor mental health and wellbeing before lockdown. However, the sample in Widnall et al. had lower proportions of Black, Asian and Minority Ethnic (BAME) students, students receiving free school meals and with a long-term illness or disability, as well as lower levels of anxiety and slightly higher school connectedness at timepoint one when compared to non-responders. A mixed picture was also presented by Demkowicz et al. (2020) who highlighted that the first national lockdown represented a time of heightened and mixed emotions for young people aged 16–19, some of which were positive, such as a relief from the normal pressures of life, but that this was in addition to stress, fear, and anxiety. In terms of differences according to gender and SES, findings from the Co-Space study (reported by parents/carers rather than young people themselves) demonstrate higher levels of behavioural and restless/attentional difficulties for secondary school aged boys than girls, but higher levels of emotional difficulties for girls than boys [51]. This study also found that, on average (throughout the pandemic), parents/carers from households with lower annual incomes (<£16,000 p.a.) reported higher levels of all behavioural, emotional, and restless/attentional difficulties than parents/carers from households with higher annual income (>£16,000 p.a.) [51].

#### 4.1.2. Young People’s Coping Mechanisms during Periods of Restriction

Day to day, young people articulated a range of coping mechanisms such as keeping busy, maintaining routines, using technology to keep in contact with friends and family and taking advantage of better weather and easing of restrictions across summer months. At a much broader, macro level, young people coped through reinforcing that sacrifices were needed in order to keep people safe. Whilst we did not set out to specifically examine this, young people organically reflected on adherence to restrictions and their frustrations with both the Government and those not following the rules, and their views on this became a dominant part of their narratives. Young people in our study articulated high levels of adherence to social distancing rules, and a sense of responsibility and burden for other people’s health. Similarly, Demkowicz et al. (2020) found that young people aged 16–19 felt they had made important sacrifices over the past year, giving up normal teenage experiences [46]; however, Murphy et al. (2020) found that survey respondents aged 14–30 appeared generally supportive of, and compliant with, lockdown measures [18].

This mirrors population-level data on adherence to rules and guidelines [52] and ONS data which reveal very high levels of adherence to social distancing and very low levels of social mixing amongst students despite misrepresentation and media tropes of irresponsible behaviour [53]. Nevertheless, this does lead us to question whether some people—young and old—have simply coped because they believed that lockdown and social distancing restrictions would be short-term. Whilst there are well-documented issues associated with terms such as ‘pandemic fatigue’, particularly where used to explain non-compliance or reinforce blame narratives [54], the sustained nature of social distancing restrictions in England means that follow-up interviews in late Autumn 2020, and initial findings from our next wave of data collection in early 2021, indicate that young people in our study are already incredibly tired and despondent, with many articulating huge differences between ‘Lockdown 3’ and previous national and local lockdowns.

#### 4.1.3. The Impact of ‘Fractured’ or ‘Interrupted’ Experiences of Schooling

Young people’s narratives were dominated by their thoughts and experiences of school closures and changes to their experience of education. At the time of writing, schools in England have re-opened on 8 March 2021 having been closed to all but children of vulnerable and key workers in England since 5 January 2021, with all school and college exams and assessments cancelled for a second year (see https://www.gov.uk/coronavirus/education-and-childcare, accessed on 10 March 2021). Such closures have become the source of much political and academic debate, and there have been conflicting findings as to whether school closures reduce community transmission of COVID-19 [55,56,57] and the extent to which the risks in a school setting are outweighed by a need to mitigate widening educational and social inequalities [58].

Our findings demonstrate acute repercussions from disrupted or fragmented schooling (missed parts of curriculum, home schooling, cancelled exams, periods of isolation). There were some gender and age patterning in young people’s accounts. Notably, those experiencing educational transition, particularly those in exam years or moving into exam years, appeared more likely to feel stress or anxiety. We have since begun collecting the next phase of diary data—whilst outside the scope of this paper, some younger participants have subsequently moved into their GCSE year. In the earlier stages of the pandemic, these young people were at home with very little school work to do. A greater workload combined with the sustained nature of social distancing requirements means that these young people are now feeling greater levels of anxiety and isolation, a finding again complimented by WEMWBS scores. Differences according to age have also been shown in wider literature. Further findings from the Co-Space study demonstrate higher levels of behavioural and restless/attentional difficulties for primary (4–10 years old) than secondary (11–17 years old) school aged children. However, they reported higher levels of emotional difficulties for secondary than primary school aged children. Between January and February 2021, parents from both age groups reported increases in behavioural, emotional, and attentional difficulties [51].

#### 4.1.4. Young People’s Worries and Hopes about Their Future

Young people’s worries and hopes about their future were fundamentally linked to their experiences of education over the past 12 months, and they articulated high levels of uncertainty and fear about the long-term impacts this would have on their life. This is supported by existing surveys of young people; the British Science Association found that young people’s (aged 14–18) biggest fear was the impact Covid-19 was going to have on education, examination, and qualifications [59]. What we do know with more certainty is that times of crisis or periods of austerity are likely to disadvantage entire cohorts/generations [60], but that this will amplify prior forms of exclusion and marginalisation [3]. In examining what they refer to as ‘Generation COVID’ (young people under the age of 25), Major et al. (2020) highlighted distinct inequalities in education provision [61]. Thus, during April, nearly three quarters (74%) of private school pupils were benefitting from full school days—nearly twice the proportion of state school pupils (38%). These inequalities were replicated in higher and further education.

#### 4.1.5. Young People’s Expectations of a Post-Pandemic ‘Normal’

Young people identified how strange the past 12 months have been for them. Despite difficulties associated with social distancing, they expressed wariness about returning to busy and crowded spaces, and felt some parts of life has resumed too quickly. They worried that this would result in further lockdowns; in hindsight, they were right to be concerned about this possibility, As we now approach the COVID-19 ‘roadmap’ for easing of restrictions in England, our intention is to explore these ideas further in subsequent phases of this project.

### 4.2. Strengths and Limitations

There are several strengths of this study. First, the findings presented here are based on phase 1 of a longitudinal qualitative study. The remaining phases of this multi-method research project comprise: (1) a second wave of diary data collection; (2) serial interviews; (3) in-depth interviews with school staff; and (4) co-production of arts-based materials in partnership with young people and creative practitioners. As a strength of the broader study, this piece of longitudinal work will ultimately span summer 2020 as well as one full year of academic schooling. This study is the first to use longitudinal diary-based methods to document young people’s thoughts and experiences of the pandemic. Drawing on Hadi and Cross, we assert that such prolonged rapport and engagement with participants enhances rigour and trustworthiness in qualitative data [44]. Use of diaries also meant that young people’s reflections were ‘date and time stamped’ [62] and we were able to explore individual journeys, in the case of phase 1, over the course of four months. During follow-up interviews, young people reflected on keeping a diary and suggested that this was an easy thing to do, that it felt private and, at times, cathartic:

“*this is the closest thing I’ve ever done to keeping a diary and to be honest its quite therapeutic!*”—Male, Aged 17, Diary Extract, 6 October 2020.

Second, our findings advance the field methodologically through use of digital technologies (Instagram, Email, Zoom) to collect diary accounts and conduct interviews. A growing literature indicates that collecting data in this way provides rich, multi-media insights into the world of young people, not readily accessed through other methods; as well as affording young people the ability to express themselves via text and creative mediums, greater freedom to talk about sensitive issues, and to exercise control over the interviewer/interviewee dynamic [37]. We used the video-conferencing platform Zoom to conduct interviews. Qualitative studies have shown this platform to be the most effective in conducting interviews due to the user friendliness, convenience and ease of creating rapport through the screen [35,36]. One thing which surprised the research team was that many young people chose to use a digital version of a more traditional, text-based diary, rather than sending photos and videos. During follow-up interviews, young people said that most often this was because they could not find a picture that conveyed how they felt better than written word; we will continue to explore the differences between text-based and visual diary extracts throughout the broader, longitudinal study.

One limitation of our study is that curation of diaries depended very much on young people’s motivation to take part, a limitation which has been acknowledged previously [63]. Some participants responded less than others meaning that, in further studies, a more structured approach to keeping in touch could be beneficial. Diary content was also youth-led. Whilst this meant young people could write about whatever was most important to them, promoting power-sharing, it also meant that there were some experiences during the pandemic that we’re still keen to learn more about, such as impacts upon sleep and further health behaviours, such as eating patterns, and the impacts upon romantic relationships (only one young female discussed her boyfriend). In response, our next phase of diary data collection has been adapted to take this into account. Thus, we have made some probing questions more specific, for example ‘have you noticed any changes to your sleep over the past year’. One of these questions will focus on romantic relationships and we will also explore this topic in serial interviews.

Another limitation is that, whilst our sample was mixed in terms of deprivation levels and we captured experiences from a diverse set of young people, with the exception of one young carer, our study lacks the voice of vulnerable or specifically marginalised young people such as the LGBTQ+ community, young people who were shielding, those at risk of homelessness, involved in the criminal justice system, BAME communities or young people living in households with substance abuse or domestic violence. Such voices have been under-represented throughout the pandemic response, and there has been a lack of data collection on protected characteristics across the board which prevents an intersectional approach.

Thus, whilst our findings suggest that many young people are disadvantaged or struggling at the moment, even those from relatively affluent families, we are cognisant that our work may only scratch the surface of the potential impact that COVID-19 will have on CYP from different backgrounds. Here, it is important to acknowledge the issue of digital exclusion or digital poverty, which is significantly influenced by income, language, literacy, culture, and ethnicity [64]. Further, like most markers of inequality, digital exclusion follows a North/South divide, and the North East is the most digitally excluded region in England, meaning there are potentially large groups of young people with little or no internet connection at home [65]. Thus, whilst young people in our study kept in touch in a range of ways, from text messaging to email to social media platforms, school closures, and the need to mobilise quickly to capture a particular snapshot in time, meant that paper-based data collection methods could not be implemented. Whilst further data from diaries and serial interviews will be collected longitudinally from the same cohort of young people, arts-based co-production work will be developed with young people, charity partners and creative practitioners in marginalised or disadvantaged communities in North East England. The first element of this work will involve presenting back our unfolding findings and exploring differential experiences of the past year.

## 5. Conclusions

To our knowledge, this is the first study to curate longitudinal qualitative diaries with young people over the course of the pandemic. Whilst CYP are largely physically unaffected by COVID-19, they are likely to experience the most long-term damage, particularly in relation to education, mental health and employment. Our findings have several implications. Our study demonstrates that young people are not ‘all in the pandemic together’ and adds to a body of evidence focusing on how COVID-19 has widened existing inequalities for this generation. Most schools have been forced to embrace virtual learning platforms leading to inevitable debates about the extent to which digital learning will be part of the ‘new normal’, leading to some young people, particularly those from disadvantaged communities, being left behind, and jeopardising the long posited ‘levelling up’ agenda from current government. Whilst some young people developed coping strategies to mitigate harm, young people in our study reported largely negative impacts upon their mental health and wellbeing, such as isolation, stress, anxiety, grief and loss, and the long-term consequences of such sustained levels of isolation remain to be seen. Despite this, some young people were indeed very happy with social distancing restrictions—particularly school closure—and it may be that schools are not the panacea to social ills, or that the pandemic represents a turning point or ‘teachable moment’ for education. Future work should explore this with different groups of young people, particularly those with lived experience of mental health difficulties, trauma or those excluded from traditional school settings. Going forward, there is a need for clear cross-government strategy on health and social inequalities, which places CYP at the heart of this plan. There is also a need for tailored, mental health support for young people, co-produced and adapted to post-pandemic life. Further, we found that even those who struggled with social distancing measures found the leap back into busy, mainstream settings difficult when restrictions eased. Thus, echoing Dewa et al. (2020), we argue that there is also a need for practical support to help young people transition out of lockdown and into the ‘new normal’.

## Figures and Tables

**Table 1:** Characteristics of Participants.

Gender		Male: 42% (*n* = 13)
		Female: 58% (*n* = 18)
Age (in years)		13: 26% (*n* = 8)
		14: 26% (*n* = 8)
		15: 23% (*n* = 7)
		16: 19% (*n* = 6)
		17: 6% (*n* = 2)
IMD Quintile (where 5 is most deprived):		1: 29% (*n* = 9)
		2: 19% (*n* = 6)
		3: 13% (*n* = 4)
		4: 13% (*n* = 4)
		5: 26% (*n* = 8)
Ethnicity:		White British: 94% (*n* = 30)
TP1 WEMWBS (Categories)		TP2 WEMWBS (Categories)
High:	10% (*n* = 3)	19% (*n* = 6)
Average	61% (*n* = 19)	45% (*n* = 14)
Possible depression	13% (*n* = 4)	13% (*n* = 4)
Probable depression	16% (*n* = 5)	23% (*n* = 7)
TP1 WEMWBS (categories):		High: 10% (*n* = 3)
		Average: 61% (*n* = 19)
		Possible Depression: 13% (*n* = 4)
		Probable Depression: 16% (*n* = 5)
TP2 WEMWBS (categories):		High: 19% (*n* = 6)
		Average: 45% (*n* = 14)
		Possible Depression: 13% (*n* = 4)
		Probable Depression: 23% (*n* = 7)

IMD = Index of Multiple Deprivation, TP = Time Point, WEMWBS = Warwick–Edinburgh Mental Wellbeing Scale.

**Table 2:** Impacts of on young people’s mental health and emotional wellbeing—sub-themes and supporting extracts.

Sub-Themes:	Supporting Extracts:
Sadness, loss and grief	*“seeing an empty high street makes me feel a bit empty…”*—Female, Aged 16 *“I miss my friends. I miss hugging them. I miss seeing them everyday. I miss our inside jokes and laughing in every science lesson because we had no clue what the teacher was talking about. I miss that freedom to have a sleepover.”*—Female, Aged 16
Loneliness and isolation	*“It’s really strange because although I’m with all my friends, I’ve never felt so lonely and I have no idea why. I’m just begging for normality to return but I know deep down that’s never going to look the same as what it used to be, no matter how hard I want it to be! I feel even less organised and less in composure than ever before, but I’m just trying to stay positive and not think about the aspect of another lockdown:)”*—Female, Aged 15
Stress, anxiety and emotional repercussions	*“I didn’t think not seeing people would have this much of a toll on my mental health.”*—Female Aged 16 *“I was always quite social before lockdown and so sometimes I get confused and frustrated when I feel like I don’t always fit in as much. Like I said before though I reckon most people are feeling a bit like this because it’s obviously quite a big transition.”*—Male, Aged 17
Exacerbation of existing mental health issues	*“Lockdown coincided with a rapid decline in my mental health. From the start of March before lockdown was officially a thing, I kind of also had this OCD breakdown, like an episode. That was very sudden and then lockdown happened and I couldn’t get that peer support from my friends…it took me a month to even text my friends to say hello. So it was really really isolating. It was really a mental health struggle. School was a distraction from that, it was really good, I could focus on work and I wouldn’t focus on the intrusive thoughts. But then when you are at home, just sat in bed, there’s nothing to do really to distract your mind.”*—Female, Aged 16
Tiredness and boredom	*“I went out with my friends today and kind of annoying since there’s nothing to do because everything is shut which is annoying since I didn’t get to see them for a long time and now we’ve not got much to do. I know it’s better for things to be closed but it’s getting boring just playing football over and over again.”*—Male, Aged 16
Coping mechanisms	*“Normally I would be busy with other clubs like Scouts or I would be seeing my friend who lives on my street but as none of this was taking place I had to try and make my own fun. Some of my main hobbies are scootering and rollerblading (I prefer the term aggressive inline skating because it sounds cooler), so usually I would go outside and practice or drag my sister out with me.”*—Male, Aged 14
Being able to socialise with people	*“It’s so much better than it would have been ten years ago because we can Face Time our friends.”*—Female, Aged 15 *We have been to the beach hut for the day with friends. Played games, played on the beach… just chilled out.”*—Male, Aged 13
Confusion and uncertainty	“*I’ve got some plans but they feel quite unreliable just because of lockdown looming.”*—Male, Aged 17 *“The whole uncertainty of this literal global pandemic is terrifying.”*—Female, Aged 16
Family conflict	*“I had a little cry because all I feel is that I always get told off and had to let it out.”*—Male, Aged 13 *My results day was extremely emotional but not all for the right reasons. However morbid it sounds my day was ruined by my dad who is extremely emotionally unstable 24/7. It really does affect your mood when you’re treading on egg shells with an angry man. He has ruined many of the significant and happy days in my life such as the birth of my half-sister, many Christmases, a couple of birthdays and now my results day…I have moved in permanently with my mum which is a breath of fresh air and I must admit that I feel significantly more relieved and more loved here. So I guess that with every cloud there’s a silver lining!”*—Female, Aged 16
Strengthened family bonds	*“My experience as a whole has been eye opening and has brought me closer to my family.”*—Male, Aged 14
School as a source of anxiety	*“I enjoyed lockdown but going back to school was stressful as I felt on edge about going back, I don’t like school or the people there.”*—Female, Aged 13
Enjoyment of lockdown	*“I’m positive I’m glad to be back at school but some days I do miss aspects of lockdown*—*I think it’s partly because I got so used to living by myself*—*or with my own thoughts if that makes sense? But now I spend the majority of my day with other people who are all talking with each other while i still feel like I’d rather spend a lot of time by myself (I think I’ve become more introverted over lockdown if that’s possible).”*—Male, Aged 17
Pandemic life as ‘surreal’	*“Everyone carries on like it’s normal, but when I look at the bigger picture of the situation, I realise it’s anything but. This reality dawns on me quite often, and its scary how quickly things have changed. Things such as wearing masks have become second nature, almost like remembering to bring a reusable bag when you go to the shops.”*—Female, Aged 16 *“Personally I think it’s strange: it all seems so…normal; of course, things aren’t completely normal, we still have to sanitise hands or carry stationery around in a plastic wallet etc but now I’m starting to vaguely remember life before the virus and how simple it felt.”*—Male, Aged 14
Expectations of post-pandemic future	*“One thing me and somebody else was talking about, was concerts, who is going to trust going to a concert again, like a proper one.”*—Female, Aged 16 *They’ll be a lot of ups and downs. A lot of things will change with the COVID stuff.”*—Male, Aged 14

**Table 3:** Disruptions and changes to education and school life—sub-themes and supporting extracts.

Sub-Themes:	Supporting Quotes:
Stress and anxiety	*“I’ve also been feeling sick most mornings before school which I think is just because I’m anxious about having to go to school.”*—Male, Aged 16 *“it’s been a stressful couple of weeks with school. As I am in year 11, they’re making us do mocks already in case they have to use the grades we get to create our real grade at the end of the year.”*—Female, Aged 15
Excitement	*“I am very glad to be back at school and back in the swing of things again. I now feel some sort of normalness now that I am socialising once again.”*—Female, Aged 16 *“…it was nice to see everyone from school again and to finally talk to my teachers.”*—Female, Aged 15
Mixed feelings	*“Woke up sad today and it happens quite a bit... the anxiety of school is starting again and my netball is finally starting again soon which is all amazing, it’s normality... it’s just I can’t remember how to act... I’m not ready to go back!!! I have little energy and it feels like we’re being thrown in the deep end. Not sure if that makes sense but hopefully it does:)”*—Female, Aged 15
Educational transitions	*“I was in Year 11 when we went into lockdown. We didn’t have to do any work because we assumed our exams weren’t going to happen. So from not learning anything in six months to learning a lot of content, it’s been strange.”*—Female, Aged 16 *“This week has made me realise how much I would have remembered, had we taken the exams. A Level content is made from the GCSE, so taking the GCSE would mean that I would be more prepared. I guess I just wasn’t prepared for how hard A Levels were going to be.”*—Female, Aged 16
Exams	*“The past few weeks leading up to GCSE results day have been quite overwhelming for me.”*—Male, Aged 16
Disrupted or fractured learning	“…*today I am actually at home as one person in our year and quite a few in our school now have the virus. This is so annoying especially since we had no idea this was going to happen until quite late last night and so many of my books are still at school. I am hoping to be back tomorrow though which is a relief. Also, recently at school all the lots of the teachers are off after two are confirmed to have the virus. This makes lessons (when we are in school) really annoying again and it means we have some cover lessons and its quite hard. Also, all of heat nine are off like our year so our school has been affected pretty badly at this point but hopefully things will get a bit better soon.”*—Female, Aged 15
Frustration about social distancing rules within school	*“I definitely think there is going to be another spike from schools re-opening, there’s no social distancing going on and the corridors are packed so closely together. If one person gets COVID then it’ll spread very quickly but people will still blame young people for the spike even though it’s not our fault, we’re being forced to go back to school.”*—Male, Aged 16 *“The social distancing the first week was great, everybody did it. But then, after that, not all teachers, some of the teachers, laid off a bit.”*—Male, Aged 14
Worries about the future	*“I don’t like to think about 6 months, 12 months, because then I’d have to be thinking about university. But you can’t think about university, is it going to be online, are we going to have mocks and stuff, that’s one big thing I think. It hasn’t hit me yet, but it is little inklings, like, you need to think about university.”*—Female, Aged 16

**Table 4:** Frustration, Burden and Responsibility—sub-themes and supporting extracts.

Sub-Themes:	Supporting Quotes:
Missed milestones	*“So today it’s my birthday…whoop I’m 17. It’s been a strange birthday, usually I’m at school with my friends and we get to have a laugh but today I’ve seen no friends in person. It’s just very strange as your birthday’s supposed to be such a special day but typing this now it’s only just hitting me that it’s actually my birthday. The once-a-year day where it’s your special day. Don’t get me wrong, I usually dislike my birthday because of all the attention I get from my outer family, but it’s still nice to see your friends to celebrate with or have a little party or sleepover with. All I’ve done today is play Minecraft, make a card for a family friend who’s recently lost her husband, get fish and chips and go on a Zoom call…”*—Female, Aged 16 *“For the past few Halloweens, I go trick or treating with my friend (the one I usually play out with) however that might not be able to happen due to restrictions in the local area. It’s painful to see since we go to school together and talk like normal anyway but I kind of agree it’s about minimising the amount of contact and staying safe.”*—Male, Aged 14
Sacrifice and responsibility	*“Recently I made the decision to keep myself, and my family and my community safe that even though I knew I was allowed to, I wasn’t going to meet anybody outside of my social bubble, like friends. Even though I know this is the right decision, sometimes I feel left out and just sad. Sometimes my friends push me to go out but then other times I know I’m doing the right thing.”*—Female, Aged 16
(Fear of Missing Out (FOMO)	*“…there was nobody I could talk to that I could say oh, are you feeling this FOMO as well. There was nobody like that. And whenever I told my mum about it, she was like what’s there to be FOMO, I think its her traditional, old mindset, you don’t need to go out with your friends, whatever.”*—Female, Aged 16
Fear of loved ones getting ill	*“Their selfish actions could literally kill someone, because they couldn’t be bothered staying 2 metres away. My mum has 4 heart conditions with 1 of them being very rare so god knows how the virus would affect her.”*—Female, Aged 16
Guilt (breaking versus adhering to rules)	*“Even though it was so much fun and it was so nice being able to see everybody, I still felt bad even though we were staying safe.”*—Female, Aged 16 *I feel kind of guilty because I know that, in the north east at least, the main increase in infection rates is thanks to my age group and people like myself. I went to town with friends for the first night since October and to be honest it wasn’t even that different to normal*—*other than encouraged hand washing, it wouldn’t take much for the virus to spread if one person had it. I feel guilty but not enough to stop going out and enjoying myself…the way I see it is as long as I don’t catch it and give it to anyone vulnerable, I can feel ok ethically.”*—Male, Aged 17
Confusion and uncertainty	*“The last couple of weeks have been very strange. I thought it was finally going back to normal. All my dance lessons went back, I could go to see my grandma and hang out with friends but now I can’t. I don’t understand why I can be with my friends all day at school but as soon as I’m off the school but in town I can’t go to Starbucks with my friend. I really hope things go back to normal soon so I can still have my birthday party with my friends and be able to see all my family at Christmas. Fingers crossed everything gets back to normal soon.”*—Female, Aged 15
Perspectives on national and local lockdowns	*“I mean it’s frustrating because it feels like we’re taking one step forward and two back, but it’s necessary to keep everyone safe so I’m trying to keep that in mind and stay positive!”*—Male, Aged 15 *“I think they’ll help for the short term, like the first wave, but it’s going to come crashing down again like a wave does. At the peak of the lockdown people will oblige with the rules for so long until they get bored or sick of staying indoors…”*—Female, Aged 16
Frustration: rules that don’t make sense	*“Frustrating that from today we can’t meet with more than 6 people even though I’m in a bubble with over 300 people at school. Rules are confusing and don’t make sense!”*—Female, Aged 15 *“The whole local lockdown for us is just stupid since we’re allowed to be packed into a class with our friends with no social distancing or masks but then we can’t go outside of school. Also restaurants and shops are still open but we can’t even go to them with our friends.”*—Male, Aged 16
Frustration: government	*“…the government in power today has proven that they are not capable of leading the country; this is due to the complete lack of planning put into basically everything regarding exams as well as the overall handling of the pandemic. I hope that when my generation come to vote, they’ll remember this year and the shockingly bad way it was handled.”*—Female, Aged 16
Frustration: those not following the rules	*“I have had multiple fallings out with one of my best friends. Because she just doesn’t understand, well, no, she understands why, obviously, but she just won’t stop going out. And I’m like, what are you missing out on, you’ll see everyone at school in a couple of weeks, I don’t know, that got me really frustrated, why can’t people just understand, sacrifice these next few months, stay inside, and you’ll see everyone back to normal.”*—Female, Aged 16 *“…many people were not following the rules like wearing face masks, washing hands, and following the arrows, on the streets, which could make things very complicated.”*—Male, Aged 14

## Data Availability

The data that support the findings of this study are available on request from the corresponding author. The data are not publicly available due to privacy or ethical restrictions.

## Supplementary Materials

The following are available online at https://www.mdpi.com/article/10.3390/ijerph18073837/s1, S1: COREQ Checklist.
